# Partial Molar Pregnancy With Normal Karyotype

**DOI:** 10.7759/cureus.30934

**Published:** 2022-10-31

**Authors:** Hussain A Al Ghadeer, Nihad Al Kishi, Kholoud H Algurini, Ahlam B Albesher, Mohammed R AlGhadeer, Ayat A Alsalman, Aisha A Bubshait, Bassil M Alkishi

**Affiliations:** 1 Paediatrics, Maternity and Children Hospital, Al-Ahsa, SAU; 2 Obstetrics and Gynaecology, Maternity and Children Hospital, Al-Ahsa, SAU; 3 Family and Community Medicine, Al-Ahsa Family Medicine Academy, Al-Ahsa, SAU; 4 Internal Medicine, King Faisal University, Al-Ahsa, SAU

**Keywords:** normal karyotype, saudi arabia, alahsa, partial mole, hydatidiform mole, gestational trophoblastic disease

## Abstract

Partial molar pregnancy results from fertilization of a haploid ovum by two sperms or duplication of one sperm, resulting in a triploid karyotype. The coexistence of partial mole with normal fetus karyotype is rare and occurs in 0.005-0.01% of all pregnancies. It is considered a challenging diagnosis. Here, we report a case of a 38-year-old primigravida diagnosed indecently at 16 weeks of gestation. She was on regular antenatal care and had partial molar pregnancy with a female fetus with diploid karyotype and no apparent malformation. This pregnancy ended with intrauterine fetal death. Histological examination of the placenta showed partial hydatidiform mole changes.

## Introduction

Gestational trophoblastic disease (GTD) is a spectrum of benign and malignant disorders that results from abnormal placental trophoblastic tissue. GTD is classified histologically as a hydatidiform mole, which is a benign form, and invasive molar pregnancy, choriocarcinoma, placental site trophoblastic tumor (PSTT), and epithelioid trophoblastic tumor (ETT), which are malignant forms [1,2]. Hydatidiform mole called molar pregnancy is categorized based on histological features and genetic abnormalities into two forms, i.e., complete mole and partial mole. In a complete molar pregnancy, an empty ovum is fertilized by two sperms or duplication of paternal haploid chromosomes. Complete mole (90%) is diploid with 46 XX karyotypes, and only paternal chromosomes are expressed. In a partial molar pregnancy, the ovum is fertilized by two sperms or duplication of paternal haploid chromosomes. Partial mole is triploid with 69 XXY, 69 XXX, and 69 XYY karyotypes, and both paternal and maternal chromosomes are expressed [3,4]. A partial mole is usually misdiagnosed during the first trimester as missed or incomplete abortion. Less than 25% of partial mole pregnancies coexist with a single viable fetus [5], with an estimated incidence an 0.005-0.01% of all pregnancies [6].

This report describes a case of partial molar pregnancy with the molar placenta and normal karyotype embryo that ended up in fetal demise at 20 weeks of gestation. Based on our knowledge, there are limited cases of this type reported in Saudi Arabia.

## Case presentation

Our patient was informed of all procedures, signed a permission form authorizing data collection for the study, and provided complete clearance for the report and case publication. The patient was a 38-year-old Saudi female, primigravida, and her medical and surgical history was unremarkable with no family history of inherited or genetic diseases. Her last menstrual cycle was on November 4, 2020, and the expected day of delivery was July 20, 2021. She presented to the Maternity and Children Hospital, Al-Ahsa. She was on regular follow-up with antenatal care (ANC) and presented at a gestational age of 16 weeks with suspecting molar features on ultrasound (US). She was referred to the perinatology unit for confirmation of molar pregnancy. US revealed an intrauterine single viable fetus with a positive heartbeat with adequate amniotic fluid volume, low-lying placenta, and fetal biometry corresponding to a gestational age of 16 weeks + three days. No obvious anomalies were detected. She was asymptomatic, with no vaginal bleeding, abdominal pain, hyperemesis, headache, features of thyroid disease, hypertension, or diabetes mellitus. On physical examination, she was vitally stable with an unremarkable finding of systemic examination.

**Figure 1:**
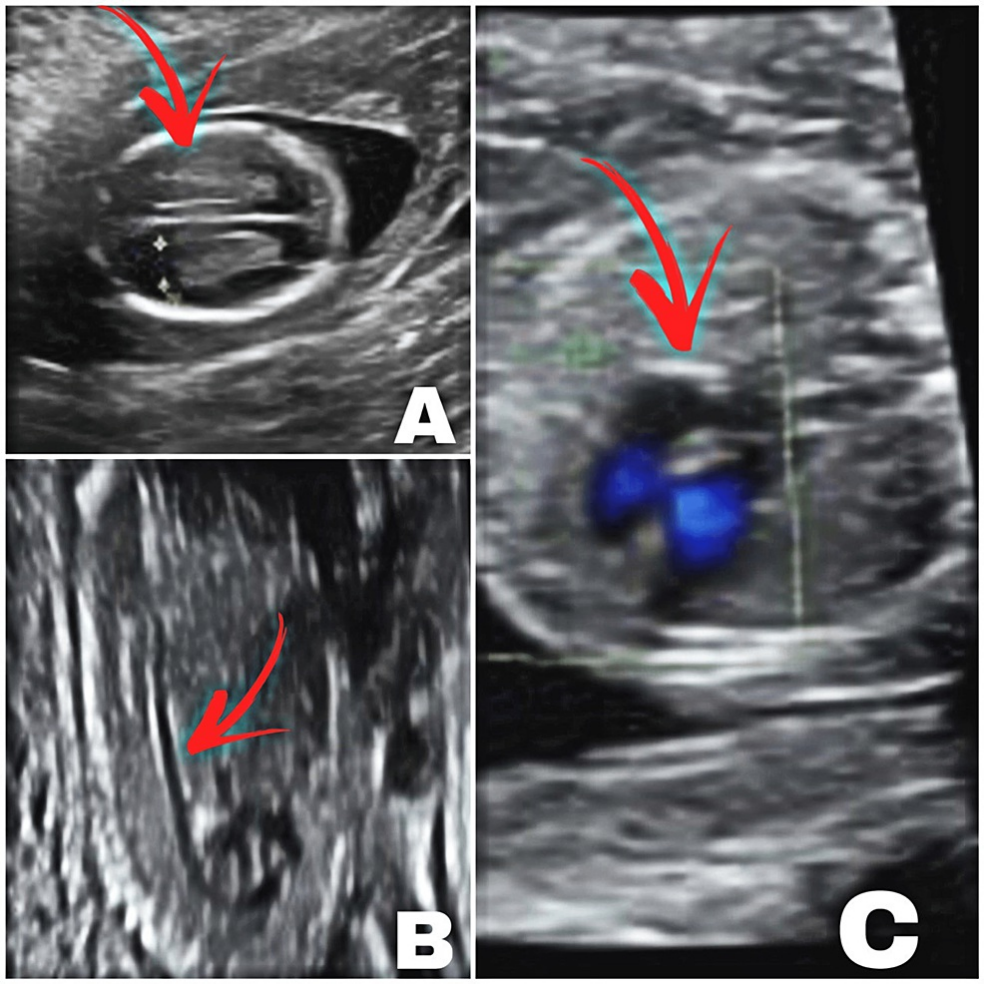
Ultrasound at 18 weeks revealed normal ventricular of the fetal head (A), with no structural or congenital anomalies in the spine (B). Four-chamber view of the heart (C)

The patient underwent amniocentesis for genetic evaluation at the gestational age of 18 weeks, which revealed a female fetus of normal karyotype 46 XX. Close antenatal surveillance was performed. She did not have any obstetrical complications during pregnancy. A repeat US at the gestational age of 18 weeks (Figure 1) revealed the fetus with a viable positive heartbeat with no significant retardation or anomalies. No evidence of placental insufficiency was noted.

**Figure 2:**
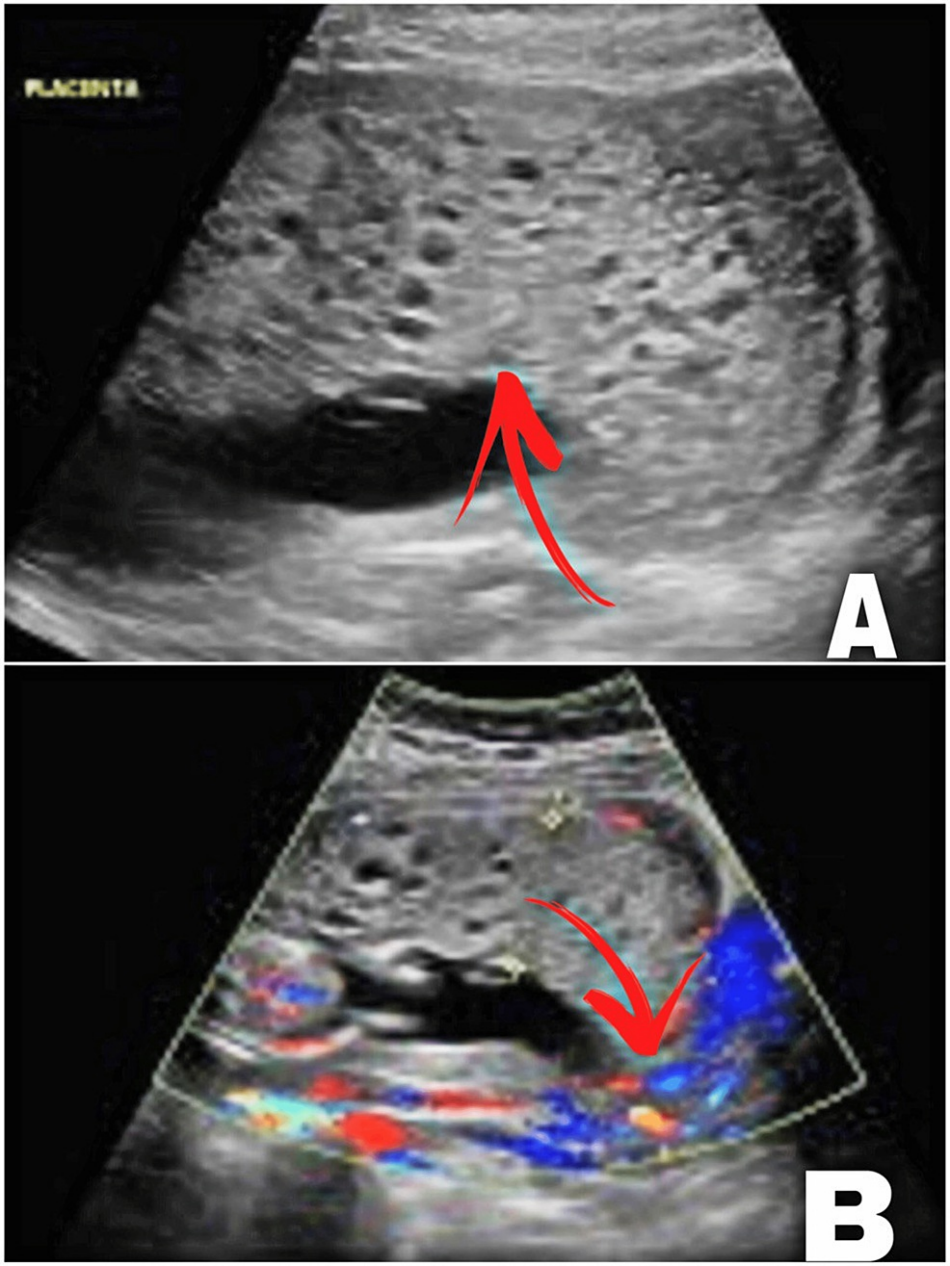
Ultrasound at 21 weeks revealed placental cystic changes

At the gestational age of 21 weeks, a scan (Figure 2) showed placental cystic changes in two-thirds of the placenta and one-third of the placental normal structure (one mass). No placentomegaly was detected and it was unlikely to be placental mesenchymal disease. The scan revealed normal fetal biometry, anatomy, adequate liquor, and no heart activity. Intrauterine fetal death (IUFD) was confirmed. At the gestational age of 22 weeks, the patient underwent dilatation and curettage. Laboratory investigation (Table 1), including complete blood count, liver, renal function tests, electrolytes, and thyroid function tests, was within normal limits. Upon admission, the serum concentration of β-human chorionic gonadotropin (β-hCG) was 1382 mIU/mL (preoperative), and post-operation, it was 800 mIU/mL. Weekly follow-up of β-hCG was done until it became undetectable. There was no evidence of persistent disease during her surveillance follow-up. The patient’s intraoperative condition was smooth. The placenta had one cord with three vessels attached to it. The placenta was sent for histopathological examination. The fetus was grossly normal and neither a visible congenital anomaly nor a feature of hydrops was witnessed. Histopathology examination revealed multiple fragments of tan-greyish to brownish grape-like spongy tissue admixed with blood clots and aggregation (10 x 10 x 8 cm). Fetal parts were not identified. Uterine contents revealed a hydatidiform molar pregnancy favoring partial mole.

**Table 1:** Laboratory investigations CBC: complete blood count; MCV: mean corpuscular volume; PT: prothrombin time; APTT: activated partial thromboplastin time; INR: international normalized ratio; NA: sodium; K: potassium; TSH: thyroid-stimulating hormone.

Laboratory investigations		
ABO	O+	
CBC	White blood cell	4.25 x 10^3^/uL
	Hemoglobin	11.3 gm/dL
	Platelets	243 x 10^3^/uL
	MCV	79.4 fL
Coagulation	PT	11.5 sec
	APTT	28.3 sec
	INR	0.96
Metabolic panel	Glucose	84 mg/dl
	Creatinine	47.55 umol/L
	Aspartate transaminase	17 U/L
	Alanine transaminase	17 U/L
	Alkaline phosphate	58 U/L
	NA	138 mmol/L
	K	4.97 mmol/L
Thyroid function test	TSH	3 mU/L
	T4	9 ug/dL
	T3	150 ng/dL

## Discussion

Molar pregnancy belongs to GTD, which results from impairment of fertilization. Molar pregnancy is divided into complete or partial moles based on genetic and histopathological features [1,2].

Complete molar pregnancy results from the parental origin with no fetal structure. About 12-20% is higher than partial mole with 4% of persistence [7]. Partial molar pregnancy results from the ovum being fertilized by two sperms, which gives triploid karyotype 69 XXY, 69 XXX, and 69 XYY. There are three types that concomitant molar pregnancy with a viable fetus. The most common type is a twin pregnancy with one normal fetus with a normal placenta and another with a complete mole. The second is a twin pregnancy with one normal fetus with a normal placenta and another with a partial mole. The third is a single viable normal fetus with a partial mole, which is considered the rarest type [8]. Our case is diagnosed with concomitant partial molar pregnancy with a normal fetus carrying a normal female karyotype that ended with IUFD.

The patient may exhibit a variety of clinical signs and symptoms of a molar pregnancy, such as vaginal bleeding, a uterus that is larger than normal for the gestational age, abdominal pain, nausea, hyperemesis, hyperthyroidism, hypertension, proteinuria, and the possibility of fetal anemia. The uterus is smaller or in line with gestational age in partial moles. Preeclampsia may indicate molar pregnancy if it is present. Consideration of the diagnosis of the hydatidiform mole should be given to very raised serial β-hCG serum concentrations coexisting with a large uterus and vaginal bleeding [9]. In this case, the patient was asymptomatic with no significant medical, surgical, or family history of a similar condition or genetic disease. In ANC and upon admission, she was hemodynamically and vitally stable with no complaints. Systemic and local examinations were unremarkable.

Although partial molar pregnancy is not routinely seen, a diagnostic workup should be established when there is a high suspicion. The majority of the cases are diagnosed in the first trimester. Ultrasound is the primary diagnostic modality of partial molar pregnancy. It reveals placental changes, placenta insufficiency, fetal malformation, abnormal growth, and oligohydramnios [10-12]. Definitive diagnosis is done by cytogenetic analysis via amniocentesis or chorionic villus sampling [12]. However, partial moles are misdiagnosed as incomplete or missed abortions [5]. Our case was diagnosed indecently in the second trimester at the gestational age of 16 weeks through routine ANC and the perinatology unit confirmed the diagnosis of partial molar pregnancy with a viable fetus. The serial US follow-up revealed a single viable fetus with positive heart activity with biometry that was proportionate to gestational age. No fetal malformation or growth retardation was detected and placental cystic changes were not observed. Cytogenetic analysis was done through amniocentesis that showed a female with normal karyotype 46 XX. At the gestational age of 21 weeks, the pregnancy ended with IUFD.

The presence of molar pregnancy with a normal fetus carries a risk to both the mother and the child. Preterm labor, mal-presentation, preterm premature rupture of membrane, chronic trophoblastic disease, and abruption are all examples of maternal complications. Abortion, congenital malformations, premature labor, severe anemia owing to placental insufficiency, and IUFD are some fetal complications [13-16]. In this case, the mother had no serious complaints before or after dilation and curettage. The possible outcomes and advice for future pregnancy and the risks were discussed with the patient. The fetus was viable with no obvious anomalies detected until it was ended with IUFD.

The management of molar pregnancy with a viable fetus depends on several factors such as the fetal karyotype, if it is triploid or diploid, the ratio of normal to molar placenta with the degree of degeneration, time of diagnosis, and presence of maternal and fetal complications. Our case was managed conservatively with the serial US until the pregnancy ended with IUFD and then evacuated through dilatation and curettage. Serial measurement of β-hCG was done weekly until it was undetectable.

## Conclusions

Partial molar pregnancy with a viable fetus is a highly unusual syndrome with a difficult diagnosis. The best way to handle a hydatidiform mole with a coexisting living fetus is yet unknown. Clinicians are encouraged to present their unique experiences so that recommendations for the care and prenatal counseling of pregnancies with partial mole and coexistent fetuses can be developed.
